# Metabolic characteristics of long-lived mice

**DOI:** 10.3389/fgene.2012.00288

**Published:** 2012-12-13

**Authors:** Andrzej Bartke, Reyhan Westbrook

**Affiliations:** Division of Geriatrics Research, Department of Internal Medicine, Southern Illinois University School of MedicineSpringfield, IL, USA

**Keywords:** growth hormone, aging, calorie restriction, dwarf mice, metabolism

## Abstract

Genetic suppression of insulin/insulin-like growth factor signaling (IIS) can extend longevity in worms, insects, and mammals. In laboratory mice, mutations with the greatest, most consistent, and best documented positive impact on lifespan are those that disrupt growth hormone (GH) release or actions. These mutations lead to major alterations in IIS but also have a variety of effects that are not directly related to the actions of insulin or insulin-like growth factor I. Long-lived GH-resistant GHR-KO mice with targeted disruption of the GH receptor gene, as well as Ames dwarf (Prop1^df^) and Snell dwarf (Pit1^dw^) mice lacking GH (along with prolactin and TSH), are diminutive in size and have major alterations in body composition and metabolic parameters including increased subcutaneous adiposity, increased relative brain weight, small liver, hypoinsulinemia, mild hypoglycemia, increased adiponectin levels and insulin sensitivity, and reduced serum lipids. Body temperature is reduced in Ames, Snell, and female GHR-KO mice. Indirect calorimetry revealed that both Ames dwarf and GHR-KO mice utilize more oxygen per gram (g) of body weight than sex- and age-matched normal animals from the same strain. They also have reduced respiratory quotient, implying greater reliance on fats, as opposed to carbohydrates, as an energy source. Differences in oxygen consumption (VO_2_) were seen in animals fed or fasted during the measurements as well as in animals that had been exposed to 30% calorie restriction or every-other-day feeding. However, at the thermoneutral temperature of 30°C, VO_2_ did not differ between GHR-KO and normal mice. Thus, the increased metabolic rate of the GHR-KO mice, at a standard animal room temperature of 23°C, is apparently related to increased energy demands for thermoregulation in these diminutive animals. We suspect that increased oxidative metabolism combined with enhanced fatty acid oxidation contribute to the extended longevity of GHR-KO mice.

## INTRODUCTION: GROWTH HORMONE-RELATED MOUSE MUTANTS

Studies of hypopituitary, growth hormone (GH) deficient, and GH-resistant mice provided evidence that deletion of GH signals can produce an impressive extension of longevity (Brown-Borg et al., 1996; Flurkey et al., 2001; Coschigano et al., 2003). Mice lacking GH or GH receptors show numerous symptoms of delayed aging, are partially protected from age-related diseases, and outlive their normal siblings by 30–65% depending on genetic background, sex, and diet composition (reviewed in Bartke, 2011; Bartke, 2012; Brown-Borg and Bartke, 2012). Candidate mechanisms linking the absence of GH signals with extension of longevity include altered expression of numerous genes related to glucose homeostasis, protein synthesis, lipogenesis, lipolysis, and energy metabolism (Tsuchiya et al., 2004; Al-Regaiey et al., 2005; Papaconstantinou et al., 2005; Masternak and Bartke, 2007). Apparently, anti-aging effects of reduced GH signaling involve metabolic adjustments of which some resemble those that mediate the effects of calorie restriction (CR) on aging and longevity (Tsuchiya et al., 2004; Al-Regaiey et al., 2005; Bonkowski et al., 2009).

In this brief review, we will discuss metabolic characteristics of GH-deficient and GH-resistant mice which are likely to represent mechanisms of their extended longevity. Metabolic characteristics of other long-lived mutants, gene knockouts and transgenics as well as phenotypes of mice from strains with different longevity are outside the scope of this article, and the reader is referred to other reviews (Brown-Borg, 2006; Chen et al., 2010; Yuan et al., 2011).

## ROLE OF IMPROVED INSULIN SIGNALING

Improved action of insulin on carbohydrate homeostasis is among the key metabolic alterations in long-lived GH-related mutants. GH receptor disrupted GHR-KO mice with profound GH resistance (Zhou et al., 1997), GH releasing hormone disrupted (GHRH-KO) mice with isolated GH deficiency (Alba and Salvatori, 2004), and hypopituitary Ames (Prop1^df^) and Snell (Pit1^dw^) dwarf mice with deficiency of GH, along with prolactin and thyrotropin (Bartke, 2011; Brown-Borg and Bartke, 2012; Bartke et al., in press), have reduced insulin levels and enhanced insulin sensitivity (Zhou et al., 1997; Bonkowski et al., 2006; Bartke, 2011; Bartke, 2012; Brown-Borg and Bartke, 2012; Spong and Bartke, unpublished). Since hypoinsulinemia promotes insulin sensitivity and *vice versa*, it could be debated which of these characteristics is primary and which is secondary. However, available evidence suggests that reduction in GH signals affects both the secretion and actions of insulin. GH and insulin-like growth factor I (IGF-I), a key mediator of GH action, promote development and secretory function of insulin-producing beta cells in the islets of Langerhans in the pancreas. Islet volume is reduced in GHR-KO mice (Guo et al., 2005), and the number of large islets is reduced in Ames dwarf mice (Parsons et al., 1995). Insulin sensitivity is negatively regulated by GH by a variety of mechanisms including reduced adiponectin levels, enhanced mammalian target of rapamycin (mTOR) signaling, and alterations in serum lipid profiles as well as ectopic fat accumulation. Many of these effects of GH on insulin signaling are mediated by enhanced inhibitory (serine) phosphorylation of insulin receptor substrate 1 (IRS-1; Aguirre et al., 2002; Ishizuka et al., 2004; Adochio et al., 2009). All of these mechanisms appear to be involved in improving insulin sensitivity in GH-related mouse mutants (Al-Regaiey et al., 2005; Wang et al., 2006; Bonkowski et al., 2009; List et al., 2011).

## ROLE OF ADIPOSE TISSUE AND ITS PRODUCTS IN THE METABOLIC PROFILE OF GH-RELATED MUTANTS

We have recently obtained evidence that enhanced insulin sensitivity of long-lived GHR-KO mice is due to the altered secretory profile of intra-abdominal (“visceral”) adipose tissue and, in particular, to enhanced adiponectin secretion by these fat depots. It is well documented that adiponectin is an important insulin sensitizer. In comparison to normal mice, GHR-KO mutants have increased levels of adiponectin in the epididymal fat and in peripheral circulation (Al-Regaiey et al., 2005; List et al., 2011). To assess the impact of altered secretory activity of visceral fat on insulin signaling, we have compared the impact of removing most of this tissue on insulin and glucose tolerance in these mutants versus normal mice. We removed as much of the epididymal and perinephric (retroperitoneal) fat pads as was possible without endangering blood supply to the testes and the adrenals. In normal mice this resulted in significant improvements in insulin and glucose tolerance (Masternak et al., 2012) as expected from previous studies in this and other species (Shi et al., 2007; Muzumdar et al., 2008). Plasma adiponectin levels were not altered, indicating that in these animals circulating adiponectin is derived primarily from subcutaneous fat, or that other fat depots readily compensate for the consequences of removing visceral fat. In sharp contrast to these findings, visceral fat removal in GHR-KO mice reduced circulating adiponectin levels and reduced, rather than enhanced, tolerance to injected insulin or glucose (Masternak et al., 2012). Apparently, visceral fat is a major source of adiponectin in these animals and visceral fat-derived adiponectin importantly contributes to or perhaps accounts for enhanced insulin sensitivity of GHR-KO mice. In addition to differences in the levels of adiponectin, the levels of interleukin 6 (IL-6), which promotes insulin resistance, are reduced in both epididymal and perinephric fat of GHR-KO as compared to normal mice (Masternak et al., 2012). Altered IL-6 levels may have also contributed to the differential impact of visceral fat removal on insulin sensitivity in GHR-KO versus normal mice.

## INTERACTIONS OF CALORIE RESTRICTION AND GH-RELATED MUTATIONS

Association of reduced insulin levels and enhanced insulin sensitivity with extension of longevity was shown in a comparison of GH-related mutants (GHR-KO, GHRH-KO, Prop1^df^, Pit1^dw^) with their normal siblings and in studies of the interaction of some of these “longevity genes” with CR (Masternak et al., 2009). Strikingly, CR improves insulin signaling in Ames dwarf mice, in which it also extends longevity (Bartke et al., 2001; Masternak et al., 2009), but has no such effect in GHR-KO mice or in GHRH-KO males in which effects of CR on longevity are absent or minimal (Bonkowski et al., 2006, 2009; Spong, Salvatori, and Bartke, unpublished). Moreover, longevity is not enhanced in transgenic mice overexpressing a GH antagonist in which insulin levels are not suppressed (Coschigano et al., 2003). It deserves emphasis that a reduction in insulin levels and enhancement of insulin sensitivity are among the most consistently observed responses to CR in different mammalian species ranging from mice and rats to non-human primates and humans (Fontana et al., 2004; Anderson and Weindruch, 2012).

In contrast to the strong association of improved insulin signaling with extended longevity in GH-related mutants, several mutations affecting events “downstream” from GH and/or IGF-I receptors are long-lived and insulin resistant (Kurosu et al., 2005; Selman et al., 2009). Further work, including examination of insulin signaling at different stages of life history will be needed to reconcile these findings but possible explanations include the well-documented opposite effects of GH and IGF-I on insulin signaling, as well as a possibility that insulin resistance may mimic some of the effects of hypoinsulinemia by protecting the cells from excessive insulin stimulation (Taguchi et al., 2007; Selman et al., 2009).

## INFLAMMATION MARKERS AND METABOLIC ADJUSTMENTS

In addition to influencing glucose homeostasis, suppression of GH signaling promotes β oxidation of fatty acids. Fatty acid oxidation is promoted by the direct or indirect actions of peroxisome proliferator activator receptor α (PPARα), PPARγ coactivator 1α (PGC1α), fibroblast growth factor 21 (FGF-21), adiponectin, and AMP-activated protein kinase (AMPK) – and GH negatively regulates the expression or activation of each of these factors (Al-Regaiey et al., 2005; Masternak and Bartke, 2007; Bonkowski et al., 2009; Louis et al., 2010). Increases in the levels of adiponectin and activation of AMPK in GH-resistant and GH-deficient animals also reduce pro-inflammatory signals by inhibiting nuclear factor kappa B (NFκB) signaling (Salminen et al., 2011; Masternak and Bartke, 2012). The resulting shift in the balance of pro- and anti-inflammatory cytokines constitutes yet another potential mechanism of enhancing insulin sensitivity (Salminen et al., 2011). Association of an altered balance of pro- and anti-inflammatory markers with shifts in carbohydrate and lipid homeostasis in long-lived GH-related mutants can thus be related to the involvement of the same mediators of GH action in the control of inflammation and metabolism.

## MITOCHONDRIAL FUNCTION AND OXIDATIVE METABOLISM

Enhanced hepatic expression of PGC1α and reduced serum lipid levels in GH-resistant mice (Al-Regaiey et al., 2005; List et al., 2011) suggest alterations in the number and function of mitochondria. PGC1α is a key regulator of mitochondrial biogenesis, and mitochondrial utilization of fatty acids as a metabolic fuel has a major impact on lipid homeostasis and circulating lipid levels.

There is little information on the number or morphology of mitochondria in long-lived GH-related mutants, while available data suggest lack of major changes in mitochondrial density in the liver or muscle of GHR-KO mice (Westbrook et al., unpublished). In Ames dwarf mice, generation of reactive oxygen species (ROS) by the skeletal muscle mitochondria is reduced, suggesting improved mitochondrial efficiency (Brown-Borg, 2006).

We are using indirect calorimetry to study the impact of GH signaling on energy metabolism. Twenty-four hour recordings of oxygen consumption and carbon dioxide output revealed that oxygen consumption (VO_2_) per gram of body weight is significantly increased and respiratory quotient (RQ) significantly reduced in Ames dwarf and GHR-KO mice (Westbrook et al., 2009).

These differences were present whether the animals were fed *ad libitum* or fasted during the recording (Westbrook et al., 2009). Moreover, similar differences between GHR-KO and normal mice were detected after exposing the animals to a prolonged period of caloric restriction or every-other-day-feeding (Westbrook et al., unpublished). Interestingly, opposite changes (reduced VO_2_ and increased RQ) were seen in giant PEPCK-bGH transgenic mice which are hypersomatotropic, hyperinsulinemic, insulin resistant, and short-lived (Bartke, 2003; Westbrook et al., 2009). The increase of VO_2_ in GHR-KO and Ames dwarf mice was apparently not due to expressing the data per unit of body mass, because differences between mutant and normal animals were, if anything, magnified when the data were recalculated per unit of lean body mass (as determined by DEXA in age- and sex-matched mice; Westbrook, 2012).

Detecting this increase in VO_2_ was not anticipated particularly in Ames dwarf mice which are hypothyroid and hypothermic and have reduced spontaneous locomotor activity (Bartke, 2011; Bartke, 2012; Brown-Borg and Bartke, 2012). Moreover, VO_2_ was reported to be reduced in Snell dwarf mice which phenotypically resemble the Ames dwarfs (Benedict and Lee, 1936). We suspected that the increase of VO_2_ in GH-related mutants could reflect increased energy expenditure for thermogenesis needed to compensate for increased heat loss. Increased radiation of heat would be expected in these diminutive animals because of the increased body surface to mass ratio. To test the validity of this explanation, we have compared VO_2_ in GHR-KO and normal mice at a thermoneutral ambient temperature of 30°C. Under these conditions, VO_2_ of the mutants greatly declined from the values measured at lower temperature and no longer differed from the normal animals (Westbrook et al., unpublished). We conclude that increased VO_2_ in long-lived dwarf mice reflects increased energy demand for thermogenesis under conditions imposed by housing at the standard animal room ambient temperature (approximately 22°C). It is an intriguing possibility that this increase in energy expenditure might contribute to slow aging and extended longevity of these mutants. Koizumi et al. (1996) reported that the beneficial impact of CR on cancer incidence and longevity in mice can be reduced or eliminated by housing the animals at a thermoneutral temperature. However, these authors suggested that the effects of thermoneutral temperature in their study were due to eliminating torpor which was a common (daily) occurrence under the conditions of fairly severe CR they employed (Koizumi et al., 1996). We very rarely observe torpor in our animals.

Since metabolic rate declines during aging, an increase in VO_2_ in long-lived mutant mice could be viewed either as a potential mechanism of extended longevity or as a “biomarker” of delayed and/or slower aging. Association of increased metabolic rate with improved life expectancy might be due to the benefits of increased uncoupling of mitochondrial electron transport from ATP production (Brand, 2000) and activation of AMPK. Reduced mTOR signaling and S6K activity in Ames dwarf and GHRKO mice (reviewed in Bartke, 2011) may provide yet another link between the regulation of aging, oxidative metabolism, and energy substrate utilization. It was recently reported that a leucine-deficient diet which suppresses hypothalamic S6KI activity produces an increase in VO_2_ per unit of body mass and a reduction in RQ; these are alterations similar to those we detected in long-lived dwarf mice (Xia et al., 2012). Examples of the association of increased VO_2_ and reduced RQ with resistance to detrimental effects of high fat diet are provided in the next section of this article.

## ALTERED USAGE OF ENERGY SUBSTRATES

In addition to demonstrating an increase in VO_2_, indirect calorimetry studies of Ames dwarf and GHR-KO mice revealed another metabolic characteristic of these long-lived animals, namely a reduction of RQ. As was the case with VO_2_, these differences were detected during both dark (active) and light (resting) parts of the 24-h period, were present in both fully fed and fasted animals, and were opposite to changes measured in short-lived giant PEPCK-GH transgenics (Westbrook et al., 2009). Reduced RQ values indicate increased reliance on fat, as opposed to carbohydrate, as a metabolic fuel and thus denote an important shift in mitochondrial function. Increased “fat burning” by mitochondria is believed to be associated with improved metabolic efficiency and reduced production of potentially harmful ROS (Lopez-Lluch et al., 2006; Ukropcova et al., 2007; Anderson and Weindruch, 2010). Similar metabolic adjustments are associated with extension of longevity in animals exposed to CR (Anderson and Weindruch, 2010). Moreover, reduced RQ and enhanced VO_2_ were associated with protection from high fat diet-induced obesity, glucose intolerance and diabetes in mice with ablated agouti-related protein (AgRP) producing neurons and in retinaldehyde dehydrogenase 1a1 knock-out mice (Joly-Amado et al., 2012; Kiefer et al., 2012). Likely mechanisms of increased β oxidation of fatty acids in GHRKO and Ames dwarf mice include increases in adiponectin levels (Al-Regaiey et al., 2005; List et al., 2011), activation of AMPK (Al-Regaiey et al., 2005), and expression of hepatic PPARα (Masternak and Bartke, 2007).

In contrast, to findings in Ames dwarf and GHR-KO mice, extended longevity in mice with fat-specific deletion of insulin receptors, as well as improvement of the metabolic profile of obese mice after gastric bypass, are associated with increases in both VO_2_ and RQ (Katic et al., 2007; Nestoridi et al., 2012). From the data that are currently available, it is difficult to determine whether the association of increased VO_2_ and reduced RQ in long-lived GH-related mutants is in any way related to the uncommon association of increased obesity with reduced insulin and increased adiponectin levels in these animals.

## SUMMARY AND RELATIONSHIP TO REGULATION OF HUMAN AGING

**FIGURE 1:**
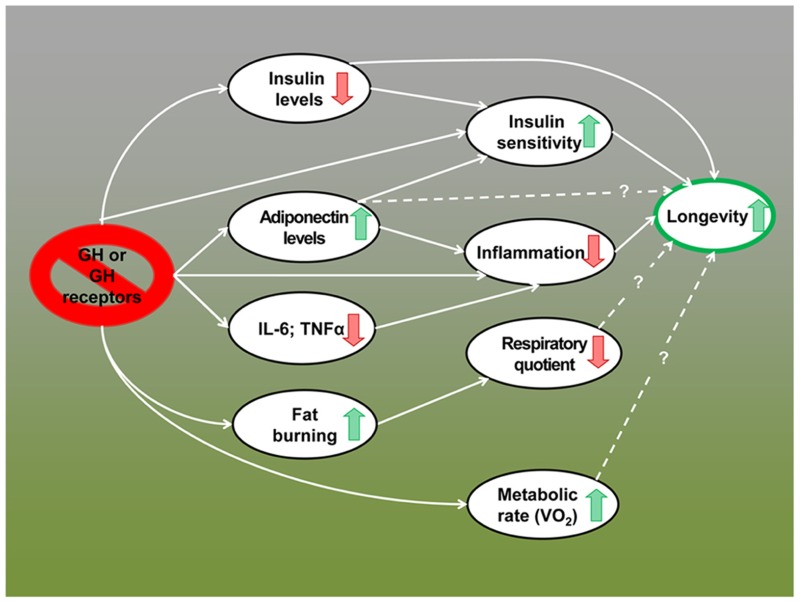
**Metabolic alterations in GH-deficient and GH-resistant mice; possible mechanisms of extended longevity**.

The remarkable extension of longevity in mice lacking GH or GH receptors appears to be due to multiple interacting mechanisms including reduced activation of growth-promoting pathways, greater stress resistance, reduced inflammation, increased reservoir of pluripotent stem cells, and improved genome maintenance (Flurkey et al., 2001; Coschigano et al., 2003; Murakami et al., 2003; Garcia et al., 2008; Bokov et al., 2009; Bartke, 2011; Ratajczak et al., 2011; Bartke, 2012; Brown-Borg and Bartke, 2012). Data summarized in this article indicate that alterations in energy metabolism and improved insulin control of carbohydrate homeostasis have to be added to this list. In fact, these metabolic adaptations may represent key features of the “longevous” phenotype of these animals and important mechanisms of the extension of both healthspan and lifespan in GH-related mutants (**Figure 1**).

Importantly, many of the metabolic features of long-lived mutant mice described in this article have been associated with extended human longevity. Comparisons between centenarians and elderly individuals from the same population and between the offspring of exceptionally long-lived people and their partners indicate that reduced insulin, improved insulin sensitivity, increased adiponectin, and reduced pro-inflammatory markers consistently correlate with improved life expectancy (Kojima et al., 2004; Atzmon et al., 2006; Baranowska et al., 2006; Bonafè and Olivieri, 2009; Rozing et al., 2011; Wijsman et al., 2011).

## Conflict of Interest Statement

The authors declare that the research was conducted in the absence of any commercial or financial relationships that could be construed as a potential conflict of interest.
